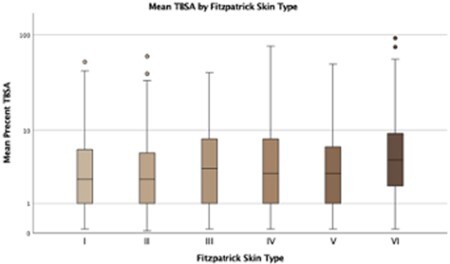# 112 The Impact of Skin Tone on Burn Interventions and Outcomes

**DOI:** 10.1093/jbcr/irae036.111

**Published:** 2024-04-17

**Authors:** Bilal Koussayer, Youssef Mohamed, Marian Mikhael, Shreya Arora, Sarah Moffitt, Nicole K Le, Kristen Whalen, Kristina Buller, Luba Ayzenshtat, Jared Troy, Jake Laun

**Affiliations:** Morsani College of Medicine USF, Tampa, FL; Morsani College of Medicine USF, Tampa, FL; Morsani College of Medicine USF, Tampa, FL; Morsani College of Medicine USF, Tampa, FL; Morsani College of Medicine USF, Tampa, FL; Morsani College of Medicine USF, Tampa, FL; Morsani College of Medicine USF, Tampa, FL; Morsani College of Medicine USF, Tampa, FL; Morsani College of Medicine USF, Tampa, FL; Morsani College of Medicine USF, Tampa, FL; Morsani College of Medicine USF, Tampa, FL

## Abstract

**Introduction:**

Historically race has been identified as a risk factor for having more severe burns, with African Americans and other minorities having a higher likelihood of experiencing worse outcomes. However, the impact of skin color on patients who get burned independent of their race is less understood.

**Methods:**

A retrospective cohort analysis was conducted on patients over the age of 18 years with burns. Patients’ skin color was classified by their Fitzpatrick skin type (FPST) using The Skin Analyzer©, which is an application that classifies patients into a FPST using images.

**Results:**

A total of 1181 patients were included in the study. On presentation there was no statistically significant difference between FPST’s burn depth, third degree burns and inhalation injury. Patients of FPST VI suffered 2.5% higher TBSA then FPST I (p=0.04). After controlling for confounding variables, FPST and race did not contribute significantly to being admitted (p>0.227). Once admitted, there was no statistically significant difference between complication rates (i.e., graft loss, regrafting, infection, and ICU days) between the FPST (p>0.05). FPST nor race significantly impacted the need for undergoing surgical intervention (p>0.517). On a generalized linear mixed model and controlling for age, TBSA, surgeries and ICU days, FPST VI had a statistically one day longer length of stay (LOS), while FPST I had a one day statically lower LOS (p < 0.001). FPST I underwent statistically more surgeries (p < 0.001).

**Conclusions:**

Our findings found a statistical differences between the TBSA and LOS in FPST groups but there is little clinical relevance to our findings. The difference in TBSA can be attributed to the easier grading of burns in darker FBST due to the stark contrast of the lost melanin in the burned area surrounded by a healthy darker skin tone. On the other hand, a difference of one day in patients LOS can be attributed due to differences in what day of the week patients were admitted as patients admitted closer to the weekend are less likely to be operated on until the following work week. Lastly, we attribute the increase in surgical intervention in FPST I patients likely due to difficultly in assessing early presenting burns as there is not a stark contrast of the burned to unburned skin. The totality of our data does not reveal any significant disparity in burn care across different FPST patients.

**Applicability of Research to Practice:**

Our findings do shed light on the notion that burn outcomes could be more likely associated with skin color rather than race, as African American patients classified outside of FPST VI did not suffer worse burn outcomes, a novel finding compared to the literature. Furthermore, Hispanics that made up a majority of the FPST III-V did not have significantly worse burn outcomes. In relation to clinical practice shows the continued importance of identifying at risk populations experiencing burns to allows us to better serve these communities.